# Facets of Nanotechnology as Seen in Food Processing, Packaging, and Preservation Industry

**DOI:** 10.1155/2015/365672

**Published:** 2015-11-03

**Authors:** Neha Pradhan, Surjit Singh, Nupur Ojha, Anamika Shrivastava, Anil Barla, Vivek Rai, Sutapa Bose

**Affiliations:** ^1^Earth and Environmental Science Research Laboratory, Department of Earth Sciences, Indian Institute of Science Education and Research Kolkata, Mohanpur, West Bengal 741 246, India; ^2^Institute of Life Sciences (An Autonomous Institute of the Department of Biotechnology), Nalco Square, Bhubaneswar, Odisha 751 023, India

## Abstract

Nanotechnology has proven its competence in almost all possible fields we are aware of. However, today nanotechnology has evolved in true sense by contributing to a very large extent to the food industry. With the growing number of mouths to feed, production of food is not adequate. It has to be preserved in order to reach to the masses on a global scale. Nanotechnology made the idea a reality by increasing the shelf life of different kinds of food materials. It is not an entirely full-proof measure; however it has brought down the extent of wastage of food due to microbial infestation. Not only fresh food but also healthier food is being designed with the help of nano-delivery systems which act as a carrier for the food supplements. There are regulations to follow however as several of them pose serious threats to the wellbeing of the population. In coming days, newer modes of safeguarding food are going to be developed with the help of nanotechnology. In this paper, an overview has been given of the different methods of food processing, packaging, and preservation techniques and the role nanotechnology plays in the food processing, packaging, and preservation industry.

## 1. Introduction

Since prehistoric age, man has been trying to improve and devise better food preservation techniques. From the cave man trying to preserve the food by storing the fresh kill in caves that provided a dampened environment in order to keep it from being spoiled to the refrigeration techniques of the 21st century, man has come a long way. Cellars and cold streams would also find their use in the preservation of food. Drying and fermentation processes existed almost 10,000 B.C. ago and today we use the modified versions of these processes [1]. Fermenting, salting, sun drying, roasting, oven baking, smoking, steaming, salting, curing, pickling, canning, bottling, jellying, irradiation, carbonation of food, and also the use of chemical or artificial preservatives are few of the methods of preservation that has been commonly used by man on a day-to-day basis. All these methods targeted at one simple idea, that is, to either slow down the multiplication of the disease causing organism or killing the organism altogether however, none of these techniques were applied with complete cognizance of the scientific mechanism behind it. Methods like osmotic inhibition and vacuum preservation are also commonly used techniques for the preservation of food. Archaeological evidence supports the idea of the practice of the mentioned preservation techniques and their existence in the Greek, Roman, and Egyptian civilizations. The Egyptians used to sun-dry their foods in order to protect them from spoilage [2]. The Romans introduced the idea of pickling in order to prevent the food from microbial infestation [3]. The Greeks were however responsible for jellying of food by introducing honey or sugar in the preservation techniques [3]. William Cullen in the year 1784 made the first technologically innovative breakthrough in the preservation techniques of food by making a crude method of artificial refrigeration [2]. The early 1800 witnessed canning of food and salting, in order to keep it fresh for a longer period of time [2]. There were others too who had made significant discoveries in the preservation techniques such as in 1809 Nicolas Appert [2] who invented a vacuum bottling technique that would supply food for French troops, and this contributed to the development of tinning and then canning by Peter Durand in 1810 [4]. With the introduction of pasteurization by Louis Pasteur in 1862, milk, wine, and beer could be preserved [4]. However, these methods were indeed crude and were unable to preserve food for a longer duration. There was a necessity of a permanent and a more reliable solution for the preservation of food.

**Figure 1:**
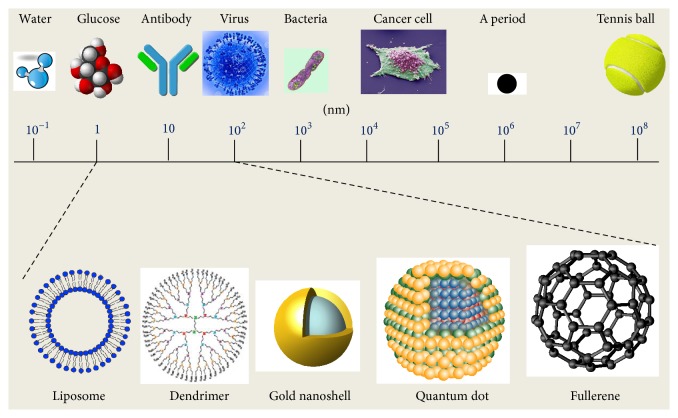
Several nanomaterials are seen, ranging from a scale of 1 to 100 nm. The reduced size of these nanomaterials makes them efficient in acting as scaffolds and aid in drug delivery. However, with nanotechnology slowly encroaching into the food sector, the services of the nanomaterials are no longer limited to the delivery of drugs, but they equally deliver food supplements, nutraceuticals [17, 18].

The word “nano” in layman terminology refers to something small, tiny, and atomic in nature [5]. The application of such an idea, incorporated with science, leads to the field of “nanoscience.” “Nanotechnology” or “nanoscience,” today, has become the call of the century. It finds its use in each and every field of science and technology. In Figure 1, a short compilation of the several nanomaterials conventionally used is given based on their size in comparison to biomolecules or microorganisms which comes close to the size of a nanomaterial. It had a revolutionary effect on the depth and pattern of our perspective. It had a thriving application in several other sectors and its application in the food industry has been a recent event. However, it has been making a steady and rapid progress in the food industry. Food quality and safety have always been a matter of great concern. Keeping the idea of a healthy population in mind, researchers have been trying to find out innovative technologies in order to improve the food quality and its safety. The intrusion of nanotechnology in the field of nutrition has led to the designing and development of novel food with better solubility, thermal stability, and oral bioavailability [6]. To incorporate functional elements in food is a field where research has been carried out for a very long time. Nanotechnology has paved the way to this idea and this has led to the development of nanoemulsions and nanocomposites [7]. Nanotechnology as such in food science is applied in several ways; it has a lot of potential that can be utilized in the improvement of the quality and safety of the food. From enhancing shelf life to improved food storage to tracking and tracing of contaminants to introduction of antibacterial or health supplements in food, nanotechnology plays a vital role in the field of food science [8].

The pre- and postharvest issues related to agricultural produce have been remarkably reduced due to the application of nanotechnology for the preservation of the food products [9]. The preservation industry has been increasing in the same rate as the food industry, almost 10–12% per year [10]. Reports have suggested that the market value of food packaging industry increased by US$2.5 billion in the year 2012 [11]. It can be further suggested based on the reports that the industry has flourished much more and the revenues have equally soared higher. Several funding bodies, such as National Nanotechnology Initiative (NNI), National Science Foundation (NSF), National Institutes of Health (NIH), Centre of Nanoscale Science and Technology (CNST), US Department of Agriculture (USDA), National Institute of Food and Agriculture (NIFA), and Agriculture and Food Research Initiative (AFRI), have been funding the research and development of nanotechnology in USA [12]. Some of the European funding bodies for nanotechnology are as follows: European Commission (EC), Engineering and Physical Sciences Research Council (EPSRC), Medical Research Council (MRC), Biotechnology and Biological Sciences Research Council (BBSRC), Regional Developmental Agencies (RDA), and Austrian Nano Initiative (ANI) [13]. As per the Indian scenario, although remarkable works related to nanotechnology are being conducted, however, the data based on their growth or even yield has not been reported. Their contribution to the food industry as such cannot be estimated currently due to lack of reported data. However, several companies have sprung up and several universities all over the country are slowly but steadily making a breakthrough in the field of nanotechnology. Several of those companies are Adnano Technologies, NanoBio Chemicals, NanoShel, NanoXpert Technologies, Sisco Research Laboratories, Quantum Corporations, DaburPharma, Meda Biotech, and Velbionanotech [14]. With funds from bodies such as Department of Science and Technology (DST), Department of Biotechnology (DBT), Department of Atomic Energy (DAE), Defense Research Development and Organization (DRDO), Indian Council of Medical Research (ICMR), Ministry of New and Renewable Energy (MNRE), and Council of Scientific and Industrial Research (CSIR), universities are also coming forward with new and innovative ways of utilizing what nanotechnology has to offer [15].

With the help of nanotechnology, the shelf life of foods can be increased and the extent of food spoilage can be decreased, as finally healthy food can reach the masses and eventually it will improve the health of the people and can aid in reducing the problem of food shortage. Several forms of “nanosystems” such as solid nanoparticles, nanofibers, nanocapsules, nanotubes, nanocomposites, nanosensors, nanobarcodes are few of the major nanomaterials that find their use in the food processing, packaging, preservation sectors [16].

## 2. Food Management

### 2.1. Food Processing

**Figure 2:**
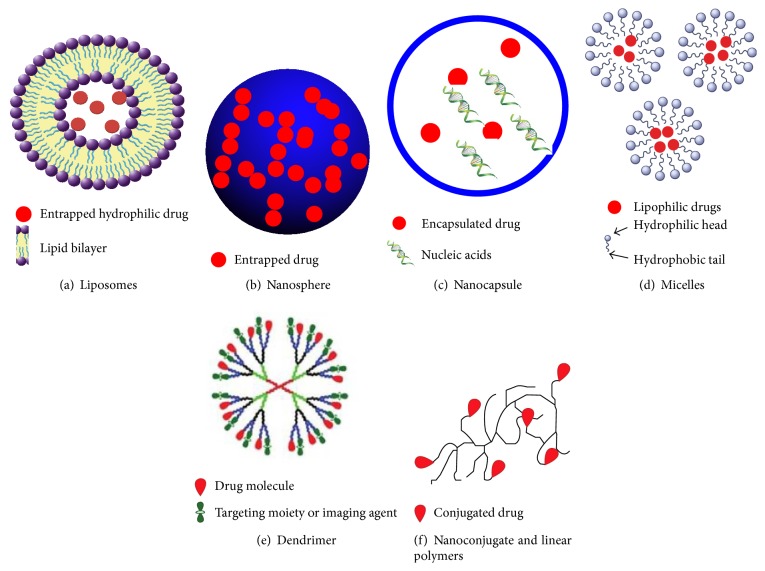
Different types of nanomaterials used in food management. (a) Liposomes (~100–400 nm) are small spherical artificial vesicles typically made with lipid bilayers. (b) Nanoparticles (~20–200 nm) are typically made with biodegradable polymers for sustained drug or antioxidants release. (c) Nanocapsules (~10–1000 nm) can encapsulate relatively large amounts of drugs and nucleic acids such as DNA, microRNA, siRNA, and shRNA. (d) Micelles (~10–100 nm) are self-assembled amphiphilic particles that can encapsulate both lipophilic or lipophobic drugs stabilized by surfactants. (e) Dendrimers (~3–20 nm) are mono-disperse macromolecules that can be used to encapsulate or covalently conjugate drugs, targeting moieties and imaging agents. (f) Nanoconjugates are polymers to which drug molecules are covalently conjugated [19].

Food processing can be defined as a practice of preserving food with the help of methods and techniques in order to transform food to a consumable state. These techniques are designed as such that the flavour and quality of the food are kept intact but they are also protected from infestation of microorganisms that leads to food spoilage. Irradiation, ohmic heating, and high hydrostatic pressure are few of the conventional methods of food processing [17, 18]. Food processing methods that involve the nanomaterials include incorporation of nutraceuticals, gelation and viscosifying agents, nutrient delivery, mineral and vitamin fortification, and nanoencapsulation of flavours [67, 68]. In Figure 2, diagrammatic examples of several nanomaterials used in food processing are summarized. Processing of food is mainly carried out in order to keep the food intact and also to increase its shelf life. Processed foods help the producer to transfer it over very large distances without running the risk of the food being spoiled. Yearly availability of different kinds of food, especially the seasonal ones such as peas or corns, is also one of the perks of processed food. Fresh foods are not the only target of food processing industry. Producing healthier food is also part of the concern and therefore these days processed food contains micronutrients which is a huge benefit for the consumers. The involvement of different nanomaterials and their techniques that find their use in the food processing industry is summarized in Table 1.

#### 2.1.1. Nanoencapsulation

Nanoencapsulation is carried out with the help of nanocapsules. They provide several benefits such as ease of handling, enhanced stability, protection against oxidation, retention of volatile ingredients, taste making, moisture triggered controlled release, pH triggered controlled release, consecutive delivery of multiple active ingredients, change in flavour character, long lasting organoleptic perception, and enhanced bioavailability and efficacy [69, 70]. They can be defined as nanovesicular systems that exhibit a typical core-shell structure in which the drug is confined to a reservoir or within a cavity surrounded by a polymer membrane or coating [21]. The cavity can contain the active substance in liquid or solid form or as a molecular dispersion. Nanocapsules are involved in the delivery of the desired component and entrapment of the odour and unwanted components in the food and thereby resulting in the preservation of the food. In the biological system, nanocapsules carry the food supplements via the gastrointestinal tract and this leads to increased bioavailability of the substance. There are six basic ways of preparation of nanocapsules, namely, nanoprecipitation, emulsion-diffusion, double emulsification, emulsion-coacervation, polymer coating, and layer-by-layer [71]. The basic difference between a conventional emulsion and nanoemulsion is that a nanoemulsion does not change the appearance of the food item when added to it. These nanocapsules find their use in the delivery of pesticides, fertilizers and vaccines to the plants. They are also used to deliver lipophilic health supplements such as vitamin and minerals in the food, fatty acids, and growth hormones, increasing the nutrient content of the food [72]. The basic benefit of encapsulation is to protect the hidden component so as to deliver it precisely at the target even in unfavourable conditions. Liposome is an example of a nanobased carrier used for nanoencapsulation. Nanoliposomes help in controlled and specific delivery of the several components within the system. They are known to deliver nutraceuticals, nutrients, enzymes, vitamins, antimicrobials, and additives [73]. Zein fibres loaded with gallic acid using electrospinning are a new method of encapsulation technique where research is being carried out [22]. Zein fibre protects the lipids from getting degraded within the system before it reaches the target delivery. This new, effective method can actually be utilized thoroughly by the food packaging industry. Lipid based encapsulation systems are much more efficient in comparison to other encapsulation systems because of the better solubility and specificity of the components encapsulated within it. This system helps the component not to interact with food material to a great extent and in this way the original characteristic of the food is kept intact and the component to be delivered within the biological system is also unaltered. Similarly, colloidosomes are hollow shell-like structures that are very minute in size almost less than a quarter of a human cell and they appear as capsules [23]. Several components are believed to be placed inside the shell and it can prove to be a good carrier of food supplements and drugs within the biological system. Likewise, nanocochleates help in improving the quality of the processed food. They are nanocoils that wrap around the micronutrients and result into stabilizing it. It is composed of soy based phospholipids which can be either phosphatidyl serine, phosphatidic acid, dioleoylphosphatidyl serine, phosphatidylinositol, phosphatidyl glycerol, phosphatidyl choline, phosphatidyl ethanolamine, diphosphatidyl glycerol, dioleoylphosphatidic acid, distearoylphosphatidylserine, dimyristoylphosphatidyl serine, and dipalmitoylphosphatidyl glycerol [2]. Nanoencapsulation of probiotics is also an emerging field where nanotechnology triumphs as it is an effort to design vaccines that will be able to regulate the immune response within the system. With the nanoencapsulation technology, the probiotics are also well preserved and delivered to the gastrointestinal tract efficiently. Starch-like nano particles help in preservation of lipid bodies and are also efficiently delivered at target site within the biological system according to several reports [74]. Archaeosomes are also an example of nanoencapsulated delivery system for antioxidants. They are prepared from archaeobacterial membrane lipids. These lipids are known to be thermostable and resistant to stress [24]. Furthermore reports have suggested that milk can be protected from degradation by nanoencapsulating *α*-tocopherol in fat droplets [70]. Few of the food products that have been commercialized that have found their use as materials for nanoencapsulation such as canola active oil, by a company called Shemen, in Haifa, Israel, which is used for the nanoencapsulation of fortified phytosterols [75]. The rest of the food products are manufactured in USA and their respective companies and product names are Fortified Fruit Juice, by a company called High Vive, which is used for the nanoencapsulation of fortified vitamin, lycopene, theanine, and sun active iron; NanoResveratrol, by a company called Life Enhancement, which is a plant-based lipid, such as a solid triglyceride, enclosed by a shell consisting of a natural phospholipid, such as phosphatidylcholine delivery system; Spray for Life Vitamin Supplements, by a company called Health Plus International, which are used for the nanoencapsulation of fortified vitamin beverage; Daily Boost by a company called Jamba Juice Hawaii, which is used for the nanoencapsulation of fortified vitamin or bioactive components beverage; Color Emulsion by, a company called Wild Flavors, which is used for the production of Beta-carotenal, apocarotenal, or paprika nanoemulsions; Nanoceuticals Slim Shake Chocolate, manufactured by RBC Life Sciences Inc. is used for the nanoencapsulation of the nano clusters that help enhance the flavour of the shake without having to add sugar to the drink [25–27, 76, 77]; and Nanotea which is another nanoencapsulated product manufactured by a company called Qinhuangdao Taiji Ring Nano-Products Co. Ltd. from China [78].

#### 2.1.2. Nanoemulsions

Nanoemulsions are used to produce food products for salad dressing, flavoured oils, sweeteners, personalized beverages, and other processed foods. They help in releasing different flavours with the help of several stimulations in the form of heat, pH, ultrasonic waves, and so forth [79]. They retain the flavours efficiently and prevent them from oxidation and enzymatic reactions. Nanoemulsions are created mainly by two approaches; high energy approach involves the steps of high pressure homogenisation, ultrasound method, high-speed liquid coaxial jets and high-speed devices method [80]. Similarly, low energy approach involves membrane emulsification, spontaneous emulsification, solvent displacement, emulsion inversion point, and phase inversion point [81]. The nanoemulsions are created by dispersing liquid phase in continuous aqueous phase. The components that are used for the creation of nanoemulsion is lipophilic where the lipophilic component is mixed thoroughly with the oil phase [28]. The placement of the lipophilic component within the nanoemulsion depends on several factors such as molecular and physicochemical properties. The physicochemical property includes hydrophobicity, surface activity, oil-water partition coefficient, solubility, and melting point [29]. Several lipophilic components are encapsulated with the help of nanoemulsion formation, for example, *β*-carotene, citral, capsaicin, flaxseed oil, tributyrin, coenzymeQ and several oil soluble vitamins [82]. They are highly stable to gravitational separation and droplet aggregation and nanoemulsion is also thermally stable in comparison to the conventional emulsions. Nanoemulsions are preferred these days rather than the conventional emulsions because the smaller the droplet is, larger the surface area is and the more readily they will be digested by the digestive enzymes and ultimately be absorbed easily. Smaller emulsions are helpful in penetrating the mucous layer present in the small intestine [83]. With the advantage of being smaller, the emulsions are transported across the epithelial layer and therefore help in better adsorption of the components that forms the emulsions. The solubility of the lipophilic components also increases when they are smaller in size [84]. Nanoemulsions in the form of proteins (e.g., egg, milk, and vegetable protein) or carbohydrates (e.g., starch, pectin, alginate, carrageenan, xanthan, and guar gum) help in improving the texture and lead to uniformity of the ice cream [85]. Brominated vegetable oil, ester gum, dammar gum and sucrose-acetate isobutyrate are used as weighting agent [86]. Weighting agents are used to reduce creaming and sedimentation. They are also known to help in the dispersion and availability of the nutrients in the food [87]. Biomolecules like milk proteins and micelles and carbohydrates such as dextrin can actually prove to be potential carrier of nutrients with the help of encapsulation [88]. Hydrolyzed milk proteins such as *α*-lactalbumin have evolved to be a potential carrier of drugs, nutrients, and supplements [89]. Casein micelles and carbohydrates such as dextrin also act as carriers. Casein micelles best serve the purpose of delivering the hydrophobic nutraceuticals [89]. Nanoemulsions are known to have antimicrobial activity and they are more effective on Gram-positive organisms than on Gram negative-organisms [90]. Due to this reason the nanoemulsions are used for decontaminating food packaging articles. Microbial growth is avoided with the help of nanoemulsions developed from nonionic surfactants, soybean oil, and tributyl phosphate [91]. Self-assembled nanoemulsions are responsible for keeping the flavour of the functional compounds from the degrading actions of enzymes, temperature, oxidation processes, and change in pH and hydrolysis processes [92]. The functional compounds that are generally encapsulated by the self-assembled nanoemulsions are lutein, *β*-carotene, lycopene, vitamins A, D, E3, and Q10, and isoflavones [93]. Nanoemulsions basically rose to fame as a delivery system of phytochemicals. Mainly two of the phytochemicals, namely, carotenoids and polyphenols, are responsible for lowering blood pressure, reducing cancer causing factors, regulate digestive tract system, strengthen immune system, regulate blood sugar level and cholesterol, and also act as antioxidants [30, 94–96]. However the only problem the manufacturers faced was the lack of proper bioavailability of these phytochemicals. Nanoemulsions made this impossible feat feasible by increasing the bioavailability of the phytochemicals by devising efficient delivery systems. The smaller is the size of lipids, the higher is the bioavailability of the phytochemicals [97]. Nanoemulsions triumph over the conventional emulsions due their reduced size. Reduced size provides larger surface area which results into increasing the rate of adsorption. This is the basic principle in making the nanoemulsions efficient.

### 2.2. Food Packaging and Food Preservation

Food packaging methods are used to make sure that the quality of the food is kept intact however; they are packaged in a way so that it is safe for consumption. Packaging mainly aims at providing physical protection in order to prevent the food from external shocks and vibration, microbial infestation, and temperature in providing barrier protection by scavenging oxygen and other spoilage causing gases. The packaging materials are preferably made of biodegradable materials in order to reduce environmental pollution. This idea has been turned into reality due to the introduction of nanotechnology in food packaging industry. High barrier plastics, introducing antimicrobials, and detection measures for contaminants are few of the methods that require being paid attention to while food is being packaged. A summary of the different type of nanotechniques used for the preservation and packaging of food is given in Table 2.

Whereas treating and handling of food in order to slow down the spoilage, resulting in the prevention of loss of food quality, edibility, or nutritive value by the microorganisms, are termed as food preservation. Drying, canning, and freezing are few of the conventional methods that have found their use as food preservation techniques.

**Figure 3:**
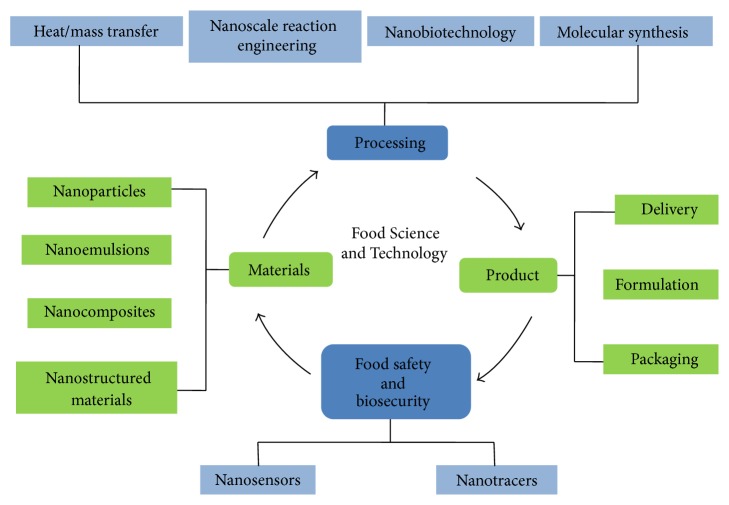
A summarized version of different steps of food management and the contribution of nanotechnology to each of the steps is given [20].

Food is managed in several different steps which involve processing, packaging and preservation means at the same time. Each of these steps is assisted by nanotechnology with the help of several nanomaterials. A flowchart in Figure 3 represents the idea.

#### 2.2.1. Nanosensors

Nanosensors help in detecting any sort of change in the colour of the food and it also helps in the detection of any gases being produced due to spoilage. The sensors are usually sensitive towards gases such as hydrogen, hydrogen sulphide, nitrogen oxides, sulphur dioxide and ammonia [31]. They are a device comprising an electronic data processing part and sensing part that is able to detect any change in light, heat, humidity, gas, and chemicals into electrical signals [98]. The high sensitivity and selectivity of the nanosensors make them more efficient than the conventional sensors. These gas sensors are made up of metals such as Palladium, platinum, and gold [99]. Gold based nanoparticles are also used at times to detect toxins such as aflatoxin B1 in milk [32]. At times they are even made up of single walled carbon nanotubes and DNA, which increases the sensitivity of the sensors. In agriculture, nanosensors help in monitoring the condition of the soil required for the growth of the crop. They also help in detecting the presence of pesticides on the surface of fruits and vegetables. Not only pesticides, but there are also nanosensors that have been developed to detect carcinogens too in food materials [33]. Gas sensors are also made up of conducting polymers. These electroactive conjugated polymer based sensors have conducting particles implanted within an insulating polymer matrix [100]. The governing factors of the conducting polymers are mainly electrical, optical, and magnetic properties and they are related to their conjugated *π* electron backbones. Change in resonance which is brought about in the presence of the gas to be detected results in a response pattern on the conducting polymer based sensor [100]. Reports suggest that these sensors have also been used for the detection of food-borne pathogens when the nanosensors were embedded with carbon black and polyaniline [34]. Nanosensors can also be installed at the packaging plant itself where they can detect the microorganisms that usually infest the food. In this way the packaged food product does not need to be sent to the lab for sampling. These sensors alert the consumers regarding the quality of the food product with the help of colour changes. The commonly used sensors that are used in the food packaging industries are time-temperature integrator and gas detector. Several different types of nanosensors are used for example, array biosensors, nanoparticle in solution, nanoparticle based sensors, electronic noses, nano-test strips, nanocantilevers [101]. Electronic noses are a type of sensor that uses several chemical sensors which is attached to a data processing system [35]. Since the sensor behaves like a human nose, the sensor is known as electronic nose. Along with the electronic nose, there are reports of electronic tongue sensors that work on a similar principle as that of an electronic nose. It changes colour on coming in contact with any sign of spoilage in the food material thus declaring that the food is not fit for consumption [36]. For the electrochemical determination of the adulterants in food and beverages such as food dyes, for example, sunset yellow and tartrazine, carbon ceramic electrode is customized with multiwalled carbon nanotubes ionic nanocomposites [102]. Biosensors are also an emerging technology which is being applied successfully. Along with the nano-gas sensors, nano-smart dust can be used to detect any sort of environmental pollution [37]. These sensors are composed of tiny wireless sensors and transponders. Nanobarcodes are also an efficient mechanism for the detection of the quality of the agricultural produce [103]. For the detection of the viruses and the bacteria, these days the nanobiosensors are proving to be quite handy and efficient. Biomimetic sensors and smart biosensors have also been reported and they are efficient in determining the presence of mycotoxins and several other toxic compounds [38]. The biomimetic sensors are developed using protein and biomimetic membranes. The sensors act as pseudo cell surfaces which help in detecting and removal of the pathogens [104]. Surface Plasmon-coupled emission biosensors modified with gold nanoparticles help in the detection of pathogenic organisms [39]. The use of nanosensors has actually helped in reducing the detection limits as they were modified to have improved level of selectivity and sensitivity. There are several immunosensors that have been developed to detect several toxins such as ochratoxin A that is detected by cerium oxide immunosensor and chitosan based nanocomposite; carbon nanotubes and silicon nanowire transistors detect* staphylococcal enterotoxin B* and* cholera toxin* [105]. Nanocomposites of SnO_2_, microrods of titanium dioxide, and SnO nanobelts are used for the detection of volatile organic compounds such as ethylene, carbon monoxide, acetone and ethanol [40]. Nanocomposites of SnO_2_ detect the presence of oxygen in packaged foods. It has to be photosensitized by UV-B radiation unlike the titanium dioxide particles which require UV-A radiation [41]. The sensor comprises an electron donor in the form of glycerol, a redox dye in the form of methylene blue, and an encapsulating polymer which is made up of hydroxyethyl cellulose. On irradiation with UV-B, the redox dye is bleached and, in the presence of oxygen, the original blue colour is seen. The extent of blue colour of the sensor is directly proportional to the presence of oxygen [106]. Sensors designed to detect the presence of pathogens in food materials provide the biggest advantage in reducing the incubation time required in conventional methods to detect the presence of pathogens. Along with all these above-mentioned sensors are indicators known as time-temperature integrators. They are mainly of three basic types, namely, abuse indicators, partial temperature history indicator, and full-temperature history indicator [42]. A time-temperature indicator/integrator helps in detecting the spoilage of food based on the history of temperature. Abuse indicators are also known as critical temperatures as they help in determining whether the desired temperature has been achieved or not [42]. Partial temperature history indicator is responsible for integrating time-temperature history when the temperature exceeds a certain predetermined value. Full-temperature history indicator registers a continuous change in temperature with respect to time [42]. Commercial availability of such TTI (time-temperature integrator) in the form of iSTrip, made by the company Timestrip, with the help of colour change, detects the change in temperature of frozen foods [42]. They are made up of gold nanoparticles which change colour to red when there is a sudden rise in freezing temperature while preserving frozen foods. Sensors known as reflective interferometry have been developed to detect* E. coli* contamination in packaged foods [43]. The protein of* E. coli* is placed on the silicon chip which binds to the similar protein in the presence of contamination. It works on the principle of scattering of light by the mitochondria. This scattered light is detected by analysing digital images.

#### 2.2.2. Nanocomposites

Nanocomposites are usually made up of polymers in combination with nanoparticles and they help to enhance the property of the polymer by combining with it [44]. Nanocomposites basically provide a highly versatile chemical functionality and therefore they are used for the development of high barrier properties [107]. They help in keeping the food products fresh, devoid of any microbial infestation for a sustainable amount of time. They usually act as gas barriers in order to minimize the leakage of carbon dioxide from the bottles of carbonated beverages [107]. In this way, it increases the shelf life of the product. Instead of making expensive cans and heavy glass bottles, manufacturing industries can use the nanocomposites to layer their bottles in order to prevent the leakage. Nanoclay is the example of a nanocomposite which is used to create these gas barriers and it is an example of a polymer in combination with nanoparticles. Nanoclays are naturally occurring aluminium silicates, generally referred to as phyllosilicates, and are inexpensive, stable, and ecofriendly in nature [45]. Phyllosilicates are found as montmorillonite, kaolinite, hectorite, and saponite based on their compositions. Nanoclay reinforcements categorize nanoclay nanocomposites into two broad categories, namely, intercalated nanocomposites and exfoliated nanocomposites [45]. Intercalated nanocomposites are ordered multilayer polymeric structure with alternating polymeric layers that are formed due to the penetration of polymer chains into the interlayer region of the clay [45]. The exfoliated nanocomposites are clay layers, randomly dispersed in the polymer matrix, and are formed due to extensive polymer penetration. Cellulose nanoreinforcements result into inexpensive, light weight nanocomposite [46]. These cellulose reinforcements are grown in plants in the form of microfibrils that are stabilized by hydrogen bonds. Such reinforcements make the nanocomposites much flexible and provide low permeability of the polymer matrix. Single walled nanotubes along with silicon dioxide nanoparticles copolymerized and gives rise to excellent gas barriers [47]. The commercial names of few of the nanoclays available in the market are Aegis, Imperm, and Durethan [47]. These nanoclays based polymers that are available in the market count above the rest due to their biodegradable nature, low density, transparency, good flow, and better surface properties. Aegis acts as oxygen scavengers and thereby improves the barrier property of the clay retaining the carbon dioxide in the carbonated drinks [47]. Durethan is made up of polyamide and it provides stiffness to the paperboard containers for fruit juices [47]. Imperm which is another example of commercialized nanoclay based polymer is made up of nylon and nanoclay and is meant to scavenge oxygen [47]. Nanocor, an example of gas barrier which is also a nanoclay based polymer, is used in the manufacturing of plastic beer bottles in order to prevent the escape of carbon dioxide from the beverage [108]. Nanocoatings for example, nanolaminates, are another example of nanoencapsulation. These nanolaminates are used to coat meats, cheese, fruits, vegetables, and baked goods. Polymers that are reinforced with metals act as antimicrobials in the form of nanozinc oxide and nanomagnesium oxide.

Zinc oxide and pediocin incorporated nanoparticles in the nanocomposite films also have antimicrobial activity [48]. Silver coated nanocomposites also act as an antimicrobial agent. Silver attaches to the cell surface and degrades the lipopolysaccharide and hence results into increased permeability, causing irreversible damage to the bacterial DNA. In order to control pests at stores that infest the packaged food materials, PEG coated with garlic oil nanocomposites prove to be very effective [48]. Bionanocomposites, which are usually made up of starch and cellulose derivatives, poly lactic acid, polycaprolactone, polyhydroxybutyrate, and polybutylene succinate, have proven to be efficient as layering materials for the packaging applications [26]. Another nanocomposite based commercialized product is known as Guard IN Fresh which helps in ripening of vegetables and fruits by scavenging ethylene gas [109]. Nanocomposites are widely used in the field of food packaging as they are known to be ecofriendly ad biodegradable. Top Screen DS13 is one such example of a nanocomposite which is easily recyclable [49]. Unlike the wax-based coating, Top Screen DS13 flaunts the idea of being water based and hence easily degraded. Another such ecofriendly nanocomposite based coating material is known as NanoCeram PAC and it helps in rapid absorption of unpleasant components which may cause foul odour and create repulsive taste [110]. Immobilization of enzymes and their use in the packaging of food is not a very widely travelled path; however, it is catching pace in the ever-evolving food industry. The method is not very popular as enzymes are sensitive to quite a number of degrading factors. Enzymes can get degraded at high temperatures or at unfavourable pH or even in the presence of proteases. Lactase or cholesterol reductase in packaging materials helps the consumers who are deficient in these enzymes in their system [50]. Enzyme immobilization triumphs over the conventional systems of coatings used in packaging materials as they provide a larger surface area and faster transfer rates. It is considered as the most effective type of nanocomposite, used in the food packaging industry. The enzymes are incorporated into the nanoclays and used for packaging of food [51].

#### 2.2.3. Nanoparticles

At nanoscale, nanoparticles serve several purposes in the processing of food. They help in improving the food's flow property, colour, and stability. The effectiveness of the nanoparticles in the food depends on its bioavailability in a system [52]. Previously, nanoparticles were used as delivery systems for drugs and now they find their use in food industry in a similar fashion. In the form of plastic films, nanoparticles, such as silicate nanoparticles, zinc oxide, and titanium oxide, are used to reduce the flow of oxygen inside the packaging containers [53]. They also help in reducing the leakage of moisture, keeping the food fresh for a longer time [53]. There are nanoparticles that aid in selective binding and hence lead to the removal of the pathogens or chemicals from food [111]. Silicon dioxide and titanium dioxide are the two most commonly used nanoparticles in food packaging. Silicon dioxide finds its use as an anticaking and a drying agent [55]. It helps in absorbing the water molecules in food, thus displaying hygroscopic application. Titanium dioxide is another nanoparticle which acts as a food colourant [56]. It is known as a photocatalytic disinfecting agent. Titanium dioxide is used as food whitener for food products such as milk, cheese, and other dairy products [56]. It finds its use as a barrier in food packaging for UV protection. Silver nanoparticles act as antibacterials and hence protect the food from microbial infestation [55]. Nanosized silver particles provide larger surface area and can be easily dispersed in food and are readily ionized and chemically active, acting as a potent antibacterial agent. Silver nanoparticles prove to be effective as antimicrobials as they have a broader spectrum of activity unlike other conventional metallic nanoparticles that act as antimicrobials [55]. Silver has been known to be quite stable since it is an element and it has been reported that it does not pose any major threat to the biological system if incorporated within limits as assigned by the FDA (Food and Drug Association) standards [55]. Being stable is definitely a perk; however silver mainly scores over the rest as an effective antimicrobial since it can penetrate through biofilms [55]. Silver mainly triumphs over the rest of the antimicrobials that are available in the market because silver can be easily incorporated into the packaging materials. Silver has also proven to have lesser propensity in making microbes resistant to it and therefore these days it is a preferable means of packaging material [55]. Silver, as per reports, infiltrates within the microbial system and disrupts the ribosomal activity and hence causes hindrance in the production of several important enzymes [55]. Silver nanoparticles are more effective as bactericide towards Gram-negative organism than Gram-positive organism as it is easier for the particles to penetrate through the thinner cell wall of the Gram-negative organism [55]. Silver nanoparticles are also known to extend the shelf life of the fruits and vegetables by absorbing and decomposing ethylene [55]. Other than the silver, zinc and titanium nanoparticles, carbon nanotubes are also used for packaging of food. However, the toxicity levels are considerably high in case of carbon nanotubes and hence the use is limited. Polymeric nanoparticles are made using polymers and surfactants, alginic acid, polylactic coglycolic acid, and chitosan and are known to be efficient delivery systems [56]. Reports also suggest the development of biopolymeric nanoparticles that proved to be bactericidal. Titanium dioxide is another nanoparticle that has been reported to have antimicrobial activity however; the usage is limited as it is photocatalyzed [112]. It is only active in the presence of ultraviolet light. It is an active bactericide against several pathogens only under UV illumination. It leads to the peroxidation of phospholipids present in the cell membrane of the bacterial cell wall. Titanium dioxide nanoparticles photosensitize the reduction of methylene blue on irradiation from UV light. Upon irradiation, the particles bleach and, only in the presence of oxygen, it changes its colour to blue. Several other reported nanoparticles that have antimicrobial activity are magnesium oxide, copper and copper oxide, zinc oxide, cadmium, selenium, telluride, chitosan, and single walled carbon nano tubes. Chitosans are responsible for binding to the negatively charged cell wall as it is positively charged [57]. This leads to increased permeability and disruption of cell wall. Inorganic nanoceramic is used in cooking oil for deep-frying food [58]. The nanoparticles in the oil keep the food crispier and increase the shelf life of the food.

## 3. Health Hazards Related to Usage of Nanotechnology in Food Processing, Packaging, and Preservation Industry

There are several products that are an outcome of the ever-evolving field of nanotechnology; they pose serious threat to the health of the population. For example, although nanoemulsions have a wide field of application in the food processing industry, however, the consumers get to pay the cost in the form of physical ailments that target them. The toxic effects of these nanoemulsions are potentially related to their size. Absorption, distribution, metabolism, and excretion of the nanoemulsion changes once the size is reduced to nanodimension [59]. Concentration, particle-size distribution, electrical charge, and interfacial characteristics are the factors that are responsible for bringing about the biological changes in the human system. Nondigestible inorganic nanoparticles and digestible organic nanoparticles have different effects on the body. Nondigestible inorganic nanoparticles include minerals and metals whereas digestible organic nanoparticles include surfactants, lipids, proteins, and carbohydrates [113]. Nanoemulsion has a shell and a core arrangement where the lipophilic component comprises the core and the shell is made up of adsorbing material. The lipophilic core is mainly made up of nonpolar components such as triacyl glycerol, diacyl glycerol, essential oils, flavour oils, mineral oils, fat substitutes, weighting agent, and fat soluble vitamins, nutraceuticals [60]. The outer shell of the nanoemulsion that encloses the lipophilic core is usually made up of surfactants, phospholipids, proteins, polysaccharides and minerals [61]. Both the core and shell have different rate and extent of digestion and adsorption in the gastrointestinal tract. Based on this idea of differential rate of digestion, the fate of the nanoemulsion can be determined; however, research still needs to be carried out in this field to have a thorough knowledge regarding the fate of the nanoemulsion within the system of the human body. Bioavailability of components within the biological system is also regarded as one of the factors that determine the toxicity of nanoemulsions. At times the bioavailability of the component within the nanoemulsion might be very low; however, the adsorption rate might be very high as the component is enclosed within the nanoemulsion. This leads to bioaccumulation of the components within the system. Consumption of nanoemulsions on a very high scale can prove to be harmful as nanoemulsions are made of surfactants and solvents which are chemical in nature [114]. Natural emulsifiers are not effective in making nanoemulsions and when these are consumed at a very high level they can have adverse effect on the biological system [62]. Although lipid based nanoemulsions score above the conventional nanoemulsions as a better mode of delivery of components within the biological systems, however, high lipid content of the nanoemulsions results into adverse effects on the body such as cardiovascular disease and obesity being a few [115].

Several nanoparticles have been reported to cause cellular damage to biological systems when they accumulate within the system. At times they also disrupt the normal working of the cellular components within the biological system because there are reports that they attach to cellular receptors of the cells of the immune system and confound them [63]. The nanoparticles also at times get coated with proteins and this leads to the degradation of the protein and hence the normal cellular mechanism is disrupted. Silver nanoparticles have been reported to have adverse effects on the human system. They affect the human lung fibroblast by reducing ATP content, increasing ROS production, and damaging mitochondria and DNA [64]. It also leads to chromosomal aberration. Hence quite positively it can be said that silver nanoparticles are genotoxic, cytotoxic, and even carcinogenic. The reduced size of the nanoparticles allow it to cross the cellular barrier and its exposure leads to the formation of free radicals in the tissues and eventually leads to oxidative damage to the cells and tissues [116]. Carbon nanotubes that are mainly used as packaging material for food usually migrate into food and can lead to toxic effects on the skin and lungs of human [65].

Not only human health but also the ecosystems are highly affected by nanomaterials that are disposed of by several manufacturing industries. These nanomaterials migrate and accumulate in the water or soil and disrupt the normal biota of that region. Nanoparticles that are washed off to the marine systems form a layer on top of the marine body and prove to be toxic to the planktons [66]. The pelagic species that feed on these planktons are likely to be affected by the toxicity caused by the nanoparticles [66]. Once the nanoparticles settle down to the floor of the marine body, the benthic species are affected by them [66]. Aluminium nanoparticles have been reported to result into inhibition of plant growth [66]. A summarized view of the implications of the nanoemulsions in the biological system is given in Table 3.

## 4. Regulations and Nanosystems

There are several regulatory bodies such as the European Food and Safety Authority (EFSA), Environmental Protection Agency (EPA), Food and Drug Administration (FDA), National Institute for Occupational Safety and Health (NIOSH), Occupational Safety and Health Administration (OSHA), US Department of Agriculture (USDA), Consumer Product Safety Commission (CPSC), and US Patent and Trademark Office (USPTO) that govern the use and application of nanosystems in food [117]. Keeping the implications in mind, these regulations have to be abided by the food industry involved in the processing, packaging, and preservation of food. European Parliament and Council Legislation are responsible for meting out the regulations on the size of the nanoparticles and it is a cause of concern from the consumer point of view [118]. A fixed size needs to be maintained in case of nanoparticles as food additives. Precautionary principle (PP) has to be adopted by the industries on a strict basis so that freely engineered nanomaterials in the food are less incorporated [119]. Only after proper study and research, the nanomaterials are to be introduced in the food items. EC Food Law Regulation has chalked out several points which need to be incorporated in the designing of nanomaterials to be used in food industry. The regulation states that the nanomaterials engineered should be free of toxic and heavy metals and also from several mycotoxins toxins [120]. European regulatory body led to the Framework 1935/2004 Regulation which states that the substances that are being incorporated in the food shall not change the inherent and organoleptic properties of the food [121]. It should remain inert and should not promote deterioration of the food and prove to have harmful effect on human health. The Regulation also states that the nanocomponents that are incorporated in the food should be first studied for dose response and the toxic effect of such components. Directive 89/107/EEC states that if some active nanocomponent meant for delivery of food supplements is being added as a packaging material for food, then it has to be first assessed as a direct food additive [122]. These regulations devised by the regulatory bodies have to be followed by one and all responsible for the production of nanotechnology based materials used in the food industry. In a country like India, these regulatory norms are sometimes not followed and the consumers land up paying a price for it and most importantly there is no specific regulatory framework that exists in India. However, such a scenario should be avoided and, on a global scale, these regulations should be abided by.

## 5. Conclusion

Nanotechnology has brought forth a revolutionary effect on the food processing and preservation industry. There are definite advantages of the technology but the drawbacks are equally prominent. Several food industry giants are paying in millions to develop nanosystems that will help preserve the food better. Care should be taken while designing newer nanosystems so that they are both environment friendly and they do not have any toxic effect on the food. Thorough testing needs to be carried out in health claims of the products that are being launched. Rather than having a chemical approach towards designing the nanosystems, research should be carried out in trying to discover natural nano-systems for the delivery of drug or health supplements through food.

Nanoparticles, being ultramicroscopic in size, are easily taken up by the cells inside the human body and can have toxic effects. The toxicity is in an enhanced fashion due to their higher bioavailability and it can also affect the immune system. Silver nanoparticles, for example, can actually make the cells resistant to any other antibacterials as the mobility of the nanoparticle within the biological is still unknown [123]. Not just silver nanoparticles, several other nanoparticles, such as titanium dioxide and zinc oxide, cause environmental pollution due to their high toxicity [124]. Eco-friendly nanoparticles need to be designed which both can serve as antibacterial and also cannot cause harm to the environment.

The smaller the size of the particles or the emulsions, the higher the threat that they pose of affecting the system with the human body. Several regulatory bodies all over the world have chalked out the regulations and standards required for the usage of nanoparticles in food. They need to be carefully followed so that the consumers do not get affected. The biggest drawback in the usage of the nanosystems in the food is that, they are still under study and have not been characterized thoroughly; therefore the extent of damage that they can actually cause to the biological systems is yet to be identified.

## 6. Future Trends

Several nanosystems are still at the stage of being developed as efficient nanocomponents to find application in the food industry. Researchers are trying to develop better and more efficient nanocarriers with increased level of bioavailability without compromising the appearance and taste of the food products in which these carriers are incorporated. The idea of “Smart Packaging” is slowly being realized and research is being carried out in developing antigen specific biomarkers used in packaging of food and also the incorporation of nanoparticles to make polymer nanocomposite films [125]. The antigen specific biomarkers will help to detect the presence of the organism responsible for the spoilage of the food material. BioSilicon, designed by pSivida, Australia, finds its use in food packaging industry [126]. It is made up of nanopores and is used for packaging of food. The speciality lies in its composition; it is made up of nanostructured silicon. RFID or Radio Frequency Identification Display is a newly engineered device that helps in swift distribution of food products that have a shorter shelf life [127]. There are transistors too that are being used based on the RFID technology. These transistors are meant for detecting even a very small change in temperature, pressure, or even pH. The sensitivity level is exceptionally high in case of these transistors. The Indian scenario is not similar to that of the developed countries. Unlike the developed countries, the university-industry interaction is not a commendable one. This is one of the reasons why the product development and commercialization sector in the field of nanotechnology is still lagging behind. However, it is slowly picking up with the trends of the world and devising newer methods and making its own contribution to this field. There is plenty of potential in the India, and it can only be harnessed properly once there is proper collaboration with the scientists at the universities and the industries. Better marketing strategies and infrastructure can help heighten the present condition [128].

## Figures and Tables

**Table 1:** List of selected nanotechniques used by different food industries for food processing.

Nanotechniques	Examples with its compositions	Used in	Advantages	References
	Nanocapsules	Food processing	Enhanced stability, protection against oxidation, and retention of volatile ingredients	[21]
			Taste making and moisture triggered controlled release	
			pH triggered controlled release	
	Nanoliposomes (zein fibres loaded with gallic acid)	Food processing	Enhanced bioavailability and efficacy	[22]
			Entrapment of the odour and unwanted components in the food	
			Delivery of enzymes, additives, vitamins, and so forth in the food	
			Delivery of pesticides, fertilizers, and vaccines to the plants	
Nanoencapsulation	Colloidosomes	Food processing	Delivery of vitamin and minerals in the food	[23]
			Increasing the nutrient content of the food	
	Nanocochleates (soy based phospholipids)	Food processing	Help in improving the quality of the processed food	[2]
	Archaeosomes (archaebacterial membrane lipids)	Food processing	Delivery system for antioxidants	[24]
	Daily Boost	Food processing	Used for the nanoencapsulation of fortified vitamin or bioactive components beverage	[25]
	Colour emulsion	Food processing	Used for the production of Beta-carotenal, apocarotenal, or paprika nanoemulsions	[26]
	Nanoceuticals Slim Shake Chocolate & Nanotea	Food processing	Used for the nanoencapsulation of the nanoclusters that help enhance the flavour of the shake without having to add sugar to the drink	[27]

Nanoemulsions	Nanoemulsions	Food processing	Produce food products for salad dressing, flavoured oils, sweeteners, personalized beverages, and other processed foods	[28]
	In the form of proteins (egg, milk, and vegetable protein) & carbohydrates (starch, pectin, alginate, carrageenan, xanthan, and guar gum)	Food processing	Help in improving the texture and uniformity of the ice creams	[29]
	Brominated vegetable oil, ester gum, dammar gum and sucrose-acetate isobutyrate	Food processing	Used as weighting agent	[30]
			Used to reduce creaming and sedimentation	[31]
			Help in the dispersion and availability of the nutrients in the food	[31]

**Table 2:** List of selected nanotechniques used by different food industries for food packaging and preservation.

Nanotechniques	Examples with composition	Used in	Advantages	References
Nanosensors	Metal based nanosensors (Palladium, platinum, and gold)	Food packaging	Detection of any sort of change in the colour of the food	[31]
			Detection of any gases being produced due to spoilage	[31]
			Detection of any change in light, heat, humidity, gas, and chemicals into electrical signals	[32]
			Detection of toxins such as aflatoxin B1 in milk	[33]
	Single walled carbon Nano tubes and DNA	Food packaging	Monitoring the condition of the soil required for the growth of the crop	[34]
			Detection of the presence of pesticides on the surface of fruits and vegetables	[34]
	Carbon black and polyaniline	Food packaging	Detection carcinogens present in the food materials	[35]
			Detection of food-borne pathogens	[36]
			Detection of the microorganisms that usually infest the food	[37]
	Array biosensors, electronic noses, nano-test strips, and nanocantilevers	Food packaging	Changes colour on coming in contact with any sign of spoilage in the food material	[37]
	Nano-smart dust	Food packaging	Detection of any sort of environmental pollution	[38]
	Nanobarcodes	Food packaging	Detection of the quality of the agricultural produce	[38]
	Nanobiosensors	Food packaging	Detection of the viruses and the bacteria	[38]
	Biomimetic sensors (protein & biomimetic membranes) and smart biosensors	Food packaging	Determination of the presence of mycotoxins and several other toxic compounds Act as pseudo cell surfaces which help in the detection and removal of the pathogens	[38]
	Surface Plasmon-coupled emission biosensors (with Au)	Food packaging	Detection of pathogenic organisms	[39]
	Cerium oxide immunosensors and chitosan based nanocomposites	Food packaging	Detection of several toxins such as ochratoxin A	[40]
	Carbon nanotubes and silicon nanowire transistors	Food packaging	Detection of staphylococcal enterotoxin B and cholera toxin	[40]
	iSTrip of time-temperature indicator/integrator	Food packaging	Detection of the spoilage of food based on the history of temperature	[41]
	Abuse indicators	Food packaging	Determination of the desired temperature has been achieved or not	[42]
	Partial temperature history indicator	Food packaging	Integration of time-temperature history when the temperature exceeds a certain pre-determined value	[42]
	Full-temperature history indicator	Food packaging	Registers a continuous change in temperature with respect to time Detection of the change in temperature of frozen foods	[43]
	Reflective interferometry	Food packaging	Detection of *E. coli* contamination in packaged foods	[43]

Nanocomposites	Nanoclay (polymer & nanoparticles)	Food packaging	Used to create gas barriers which minimize the leakage of carbon dioxide from the bottles of carbonated beverages	[44]
	Aegis	Food packaging	Act as oxygen scavengers, retaining the carbon dioxide in the carbonated drinks	[44]
	Durethan (polyamide)	Food packaging	Provides stiffness to the paperboard containers for fruit juices	[45]
	Imperm (nylon)	Food packaging	Meant to scavenge oxygen	[46]
	Nanocor	Food packaging	Used in the manufacturing of plastic beer bottles in order to prevent the escape of carbon dioxide from the beverage	[47]
	Nanoencapsulation (nanolaminates)	Food packaging	Used to coat meats, cheese, fruits, vegetables, and baked goods	[47]
	Zinc oxide and pediocin & silver coated nanocomposites	Food packaging	Act as an antimicrobial agent	[48]
			Degrade the lipopolysaccharide	[26]
			Cause irreversible damage to the bacterial DNA	[49]
	PEG coated with garlic oil nanocomposites	Food packaging	Control pests at stores that infest the packaged food materials	[49]
	Bionanocomposites (cellulose & starch)	Food packaging	Proven to be efficient as layering materials for the packaging applications	[50]
	Enzyme immobilization	Food packaging	Provides a larger surface area and faster transfer rates	[51]
	Top Screen DS13 & Guard IN Fresh	Food packaging	Help in ripening of vegetables and fruits by scavenging ethylene gas	[51]
	NanoCeram PAC	Food packaging	Helps in rapid absorption of unpleasant components which may cause foul odour and create repulsive taste	[51]

Nanoparticles	Silicon dioxide	Food packaging & preservation	Reducing the leakage of moisture	[52]
			Anticaking and drying agent	[53]
			Absorbs the water molecules in food, showing hygroscopic application	[54]
	Titanium dioxide	Food packaging & preservation	Acts as a food colourant	[54]
			Photocatalytic disinfecting agent	[55]
			Used as food whitener for food products such as milk, cheese, and other dairy products	[56]
	Zinc oxide	Food packaging & preservation	Reduce the flow of oxygen inside the packaging containers	[57]
	Silver nanoparticles	Food packaging & preservation	Act as antibacterial agent and protect the food from microbial infestation	[57]
			Extend the shelf life of the fruits and vegetables by absorbing and decomposing ethylene	[58]
	Inorganic nanoceramic	Food packaging & preservation	Used in cooking oil for deep-frying food	[58]
	Polymeric nanoparticles	Food packaging & preservation	Known to be efficient delivery systems and are bactericidal	[58]

**Table 3:** Effect of nanoemulsion in human system.

Nanoemulsion	Hazardous components	Advantages	Health Hazards	References
Nondigestible inorganic nanoparticles	Silver nanoparticles	Food packaging	Reducing ATP content	[59]
			Increasing ROS production	[59]
		Food processing	Damaging mitochondria and DNA	[60]
		Food preservation	Chromosomal aberration	[61]
			Genotoxic	[62]
			Cytotoxic	[62]
			Carcinogenic	[63]

Digestible organic nanoparticles	Surfactants, lipids, proteins, and carbohydrates	Food packaging	Bioaccumulation	[64]
			Cellular damage	[65]
		Food processing	Degradation of proteins	[65]
			Cardiovascular diseases	[65]
		Food preservation	Obesity	[65]
	Carbon nanotubes	Food packaging	Cause skin and lungs disease	[66]
